# Mating Conditions and Management Practices Influence Pregnancy Scanning Outcomes Differently between Ewe Breeds

**DOI:** 10.3390/ani12212908

**Published:** 2022-10-24

**Authors:** Amy L. Bates, Shawn R. McGrath, Susan M. Robertson, Gordon Refshauge

**Affiliations:** 1 School of Agricultural, Environmental and Veterinary Sciences, Charles Sturt University, Wagga Wagga, NSW 2678, Australia; 2 Gulbali Institute, Charles Sturt University, Wagga Wagga, NSW 2678, Australia; 3 New South Wales Department of Primary Industries, Cowra, NSW 2794, Australia

**Keywords:** pregnancy scanning, mating condition score, mating liveweight, ram percentage, Bayesian network, sheep, flock management

## Abstract

**Simple Summary:**

The conditions and management practices imposed during mating can influence the success of a sheep production enterprise. However, the impact of mating practices across southern Australian sheep production systems are relatively unknown. Mating liveweight and body condition score data were collected at mating from four sheep breeds and during three seasons of mating across southern Australia. Further, the seasonal conditions during mating were ranked by the producers and ram percentage and region were also recorded. Bayesian Network analysis was used to explore the relationships between these variables and pregnancy and fetal number at scanning. The results of this survey study emphasize the interrelatedness of the explored mating conditions and practices and the importance of understanding their interactions for optimizing sheep reproduction and nutrition from mating.

**Abstract:**

Sheep production in southern Australia may vary by breed, time of year, production output (wool, meat, or both), region and seasonal influence. Sheep producers with flocks of approximately 300–500 ewes (*n* = 58) were recruited across southern Australia to take part in a survey and mating variables were collected from over 30,000 ewes between October 2020 and August 2021. A Bayesian Network (BN) was developed to identify the interrelatedness and most influential variable on pregnancy and fetal number (of pregnant ewes) outcomes under different scenarios. The BN analysis indicated a low association between the variables explored, however, were breed dependent. In wool-based breeds a mating liveweight of 60–69.5 kg predicted the lowest non-pregnant and greatest number of fetuses, and in shedding ewes 70–79.5 kg predicted the lowest non-pregnant rate and 90–99.5 kg the greatest number of fetuses. Pregnancy rate and fetuses per ewe were optimized at ram percentages of 1.5% for Composite and Merino ewes and 2% for Maternal ewes. A mating BCS 4 resulted in greatest pregnancy rate and number of fetuses across all breeds. Curvilinear relationships between mating liveweight, BCS and ram percentage were observed with pregnancy rate and fetal number. Practically, reproductive potential is best managed on a breed basis and with consideration of all variables explored.

## 1. Introduction

Sheep have a seasonal reproductive cycle, becoming most active as photoperiod shortens and experience anestrus or low ovarian activity as photoperiod lengthens [1,2]. Further, the liveweight at which optimal fertility is achieved is influenced by both mating season and breed [3,4]. Adding to variability, breeding season length [5,6,7] and ovulation rate are dependent on breed [8,9]. Mating season, liveweight and body condition score (BCS) have also been associated with variation in ovulation rate [10]. Further, the response to photoperiod length is also influenced by nutrition [11] and improving nutrition to increase liveweight or BCS prior to mating is known to improve reproductive performance [4,12]. However, a breed dependent upper liveweight and BCS bounds have also been identified, above which no reproductive advantage was achieved [13,14,15].

The management of different sheep breeds provided with the same resources may result in different liveweight gain and productivity due to the different energy requirements of larger versus smaller animals [16,17]. This is reflected in the predictability of wool production and reproductive performance based on maternal liveweight and BCS, which varies by breed [12,18]. As such, guidelines for ewe management in Australia currently have two forms, those for Merino ewes and those for non-Merino ewes [19,20].

Computer modelling of whole sheep enterprises across different locations indicated the optimum lambing time was dictated by the average length of the pasture growing season and production output (wool and/or lamb target weight) [21]. This was supported by a survey of sheep producers that identified over 53% of producers use feed availability as the primary determinant of lambing time [22], and thus month of mating. This decision may also be influenced by environmental factors, such as grass seed burden, weather extremes and the marketability of lambs.

Management conditions and practices leading up to and at mating, including ewe breed, mating season, and management of liveweight and BCS may influence reproductive performance. While guidelines have been developed, the impact of mating conditions and practices in a commercial setting is relatively unknown. The objective of this study was to examine relationships between mating liveweight and BCS on pregnancy and fetal number at pregnancy scanning across different breeds and seasons of mating in sheep enterprises in southern Australia. It was hypothesized that the relationship between mating liveweight and BCS and pregnancy and fetal number would vary between ewe breed and mating season. The impact of other variables and practices were also explored.

## 2. Materials and Methods

### 2.1. Experimental Sites and Protocols

Commercial farms within New South Wales (NSW), Victoria (VIC) and South Australia (SA) were established as experimental sites between October 2020 and May 2021, totaling 56 sites, 58 ewe flocks and 30,030 individual ewe records. Producers were recruited to the project through professional networks across a broad range of regions. These regions represent Central NSW, Northern NSW/Queensland (QLD), the Wimmera Malley Murray, East VIC and the SA Peninsula.

Each site joined flocks of either Composite, Maternal, Merino or shedding ewes during either spring, summer, or autumn (2020 to 2021) according to individual routine management practices. Ewes were individually identifiable by electronic identification tags and between 300 and 500 mature (having had at least one previous mating event) ewes were mated in each flock. The Composite hybrid animal, the result of crosses of multiple breeds, has low wool value and are primarily meat-producing animals, excluding shedding breeds. Maternal ewes are defined as meat-producing crossbred ewes which retain some wool value. Merino ewes are defined as those of various Merino genotypes with high wool value. Shedding breeds have no wool value due to a shedding hair-based coat and are solely meat-producing animals, and included Dorper and Australian White breeds.

The liveweight and BCS measurements were taken when rams were introduced to each flock to ensure accurate mating information was collected across the study. For spring mating, one Composite flock and one Merino flock used a melatonin-based hormone product (Regulin^®^, Ceva Animal Health Pty Ltd., Glenorie, NSW, Australia) to enhance reproductive performance [23]. Liveweight and BCS measurements were collected and performed by a single assessor to avoid inter-operator variability, and a single set of calibrated scales were used across the study. Although there is known association between liveweight and BCS, this may vary by parity, interbreeding interval, or breed [24] and as such, both liveweight and BCS of each individual ewe was collected. Ewes were mated in accordance with the routine practices of each individual enterprise. The assessor collected information on ram percentage used for each flock and seasonal conditions during mating. 

Pregnancy scanning was performed during mid pregnancy using transabdominal ultra-sound by various commercial scanning operators to detect fetal number in accordance with each site’s routine management practices. Not all sites identified triplets and higher order litters and as such fetal number was recorded as either non-pregnant (no detectable fetus), single (one) or multiple (two or more) fetuses. In two instances the data for multiple fetuses (one summer joined Composite flock and one autumn joined Maternal flock) was removed from the data set due to inaccurate scanning performance or because of a delayed date of scanning greater than 100 days from the start of mating. For these two sites, pregnancy status (pregnant or non-pregnant) was retained.

### 2.2. Measurements

Ewe liveweight (*n* = 29,270) and BCS (*n* = 30,030) was recorded at the commencement of mating. Seven-hundred and sixty liveweight records were missing from the dataset due to unavoidable recording errors while collecting data on commercial farms. Liveweight was recorded to 0.5 kg accuracy level, with a mean liveweight of 67.7 ± 13.4 kg across the survey. Body condition score was performed by a single assessor across the survey and recorded at quarter intervals, according to Jefferies [25] and Russel et al. [26]. The mean BCS was 3.38 ± 0.8 and ranged from 1–5 and within-flock BCS ranged by two whole scores for all flocks. Pregnancy scanning occurred approximately 70–100 days after ram introduction. The number of rams used per ewe flock was recorded as a percentage. Seasonal conditions during mating were ranked by each producer on a five-point scale between ‘well below average’ to ‘well above average’.

### 2.3. Statistical Analysis

Data were collected and imported into Microsoft Excel [27]. Descriptive and exploratory statistical data analyses were conducted in R [28].

### 2.4. Bayesian Network Model

Bayesian Network (BN) analysis was used to explore the interrelationships of the variables. The BN model was developed using the Netica software package [29]. A BN model is a graphical representation of joint probability distribution of all the variables in the model and the variables are represented by nodes which are linked based on probabilistic dependency between two associated variables [30]. The mathematical formula of the Bayes Theorem, which is the theoretic foundation of the BN approach, is given in Equation (1) [31]:

(1) $$\mathrm{Pr}\;\left(B|A\right)=\frac{\mathrm{Pr}\;(A|B)\mathrm{Pr}\;\left(B\right)}{\mathrm{Pr}\;\left(A\right)}=\frac{\mathrm{Pr}\;\left(A,B\right)}{\mathrm{Pr}\;\left(A\right)}$$

where *A* and *B* are two random variables; Pr (*A*) and Pr (*B*) are the marginal probability distributions of *A* and *B*, respectively; Pr (*B*|*A*) is the conditional probability distribution of *B* given *A*; Pr (*A*|*B*) is the conditional probability of *A* given *B*; and Pr (*A*,*B*) is the joint probability distribution for *A* and *B*. Since a BN model represents a joint probability of the variables, inferential analysis may be performed by fixing the values of a set of selected variables (similar to fixing the values of those predictor/independent variables in a regression model), then the values of remaining variables (equivalent to the response/dependent variable) in the BN model can be predicted/estimated [32].

**Figure 1:**
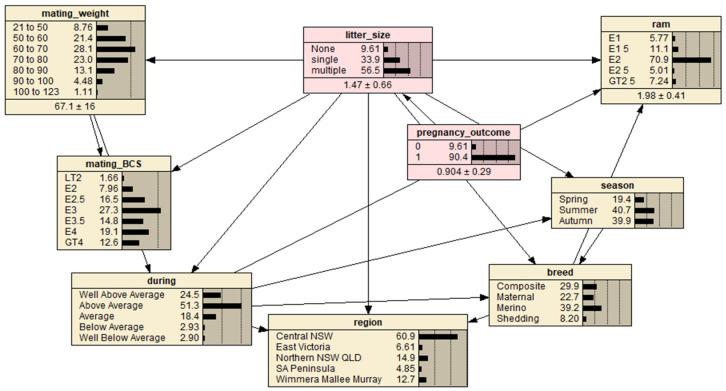
A predictive Bayesian Network model for the full ewe dataset representing interrelationships between ewe pregnancy outcome (‘0’ defined as a non-detectable fetus and ‘1’ as at least one detectable fetus) and fetal number (‘litter size’) calculated for four breeds, seven mating BCS and mating weight levels, three mating seasons, five broad regions, five subjective producer described seasonal conditions during mating and ram percentage utilized at mating. Letter prefixes of levels within nodes are defined as: LT = less than, E = a range of values around the displayed value and GT = greater than. Region abbreviations are defined as: NSW = New South Wales, VIC = Victoria, QLD = Queensland and SA = South Australia.

The most influential variable in determining pregnancy outcome (pregnant or non-pregnant) and fetal number (single or multiple fetus), respectively, could be investigated using a BN model with respect to the potential variables of breed, mating BCS, mating liveweight (weight), mating season (season), region, seasonal conditions during mating (during) and ram percentage (ram) (Figure 1). Mating liveweight, BCS and ram percentage categorical data was refined. By selecting different potential variables, the BN model estimated/predicted the likelihood of pregnancy outcome and fetal number (of pregnant ewes). 

Sensitivity to findings is an inbuilt Netica function that enables the ranking of impacts of other variables (‘evidence variables’) on a selected target variable [32]. The sensitivity analysis outcomes were presented in a table with a list of Mutual information and percentages values (ranked from highest to lowest) represent the strength of association between those evidence variables and the target variable [29]. Mutual information is a measure of information shared between two random variables that quantifies the change in uncertainty on one provided the uncertainty of the other variable is known [33]. The percentage values from the sensitivity analyses for a selected variable are broadly analogous to the adjusted R^2^, the goodness-of-fit measure from a regression analysis that represent the proportion of the variation explained in the selected variable by fixing the value in one of the other variables. A sensitivity analysis was performed for pregnancy outcome and number of fetuses (of positive (‘1’) pregnancy outcomes), respectively, to quantify the strength of the association between the interrelated variables breed, mating BCS, mating liveweight, mating season, region, seasonal conditions during mating and ram percentage. The sensitivity analysis output percentage (%) values are similar measures to the goodness of fit measure adjusted R^2^ used in a regression model, which represents the proportion of variation in the response variable being explained by those predictor variables.

## 3. Results

### 3.1. Descriptive Analysis

The majority of data was collected in the Central NSW (*n* = 18,293) region followed by Northern NSW/QLD (*n* = 4481), Wimmera Malley Murray (*n* = 3816), East VIC (*n* = 1984) and SA Peninsula (*n* = 1456) regions. A total of 58 ewe flocks were recorded across three mating seasons; spring, summer and autumn. The majority of flocks were Merino followed by Composite, Maternal and shedding ewes (Table 1). The spring-mated Composite flock and one autumn-mated Composite flock were a highly fecund sheep breed with known genes selected for increased litter size (http://www.multimeat.com.au/ (accessed on 21 August 2022)).

The heaviest average mating liveweight by breed was Maternal ewes (71.34 ± 12.1 kg), followed by Composite (71.0 ± 11.4 kg), shedding (68.8 ± 13.2 kg) then Merino (62.6 ± 14.0 kg) ewes. The highest average mating BCS by breed was shedding ewes (BCS 3.72 ± 0.7) followed by Maternal (BCS 3.62 ± 0.9), Composite (BCS 3.51 ± 0.8) and Merino (BCS 3.07 ± 0.7) ewes. 

The number of ewes surveyed by breed and season, and pregnancy scanning information is reported in Table 1. Pregnancy rates were highest in Composite ewes (94.1%), then Merino (89.5%), Maternal (88.8%) and lowest in shedding ewes (85.0%). Multiple scanned fetuses were greatest in Composite ewes (78.7%) followed by Maternal (66.8%), shedding (55.2%) and Merino ewes (47.5%).

### 3.2. Bayesian Network Model

Based on the sample data, by first excluding the pregnancy outcome variable and designating the fetal number as the target variable, the Netica inbuilt Tree Augmented Naïve Bayes Net algorithm was employed to specify a BN model structure. The pregnancy outcome was then manually added into the model as the parent node of the fetal number node. Finally, the BN model was completed by employing the inbuild Expectation-Maximization algorithm for model parameter estimation. Thus, the resultant BN model should have the best prediction performance given the sample data. However, the BN model was further refined based on a priori knowledge about the data by manually adding three links between associated variables: (1) link between ‘season’ and ‘breed’; (2) link between ‘breed’ and ‘region’; and (3) link between ‘breed’ and ‘ram’. The resultant BN model is presented in Figure 1. 

The sensitivity analyses (sensitivity to findings) for pregnancy outcome and fetal number of pregnant ewes (pregnancy outcome ‘1’ selected) allowed a quantitative comparison of the variables breed, mating BCS, mating weight, mating season, region, seasonal conditions during mating and ram percentage to determine the most influential variable (Table 2). Overall, the Netica sensitivity to findings function found that the variables explored explained little of the predicted pregnancy and fetal number outcomes. Pregnancy outcome and fetal number of pregnant ewes were each most influenced by ewe breed given the absolute influence level being very low. A series of subsequent sensitivity analyses were performed for pregnancy and fetal number of pregnant ewes of each breed to determine which variables was most influential (Table 3). Seasonal conditions during mating were most influential on pregnancy outcome for Composite and shedding ewes, while region was most influential for Maternal ewes and mating season for Merino ewes. Mating liveweight was most influential on fetal number of pregnant Composite, Maternal and Merino ewes while seasonal conditions during mating were most influential on shedding ewe breeds.

The model predicted relationship between Composite ewe fetal number and each level within each node/variable is displayed in Table 4. As seasonal conditions improved above ‘average’ non-pregnant rate also increased, as identified by the sensitivity analysis (Table 3). ‘Above average’ seasonal conditions increased fetal number, but ‘well above average’ conditions decreased fetal number. A mating BCS 4, mating liveweight of 60–70 kg and ram percentage of 1.5% resulted in the lowest non-pregnant rates and highest fetal number. Mating in spring resulted in superior reproductive potential compared to autumn and then summer. Multiple rate did not appear to be regionally dependent. Optimum reproductive potential, defined as lowest non-pregnant and highest multiple fetus rate, of Composite ewes across region and seasonal conditions was predicted to occur with a summer mating (without hormone manipulation), 1.5% ram percentage, mating BCS 4 and mating liveweight of 60–70 kg.

The model predicted relationship between Maternal ewe fetal number and each level within each node/variable is displayed in Table 5. A ‘well above average’ season predicted the least non-pregnant ewes and greatest fetal number, however ‘above average’ produced more non-pregnant ewes and fewer fetuses compared to an ‘average’ season. A mating BCS 4, summer mating, mating liveweight of 60–70 kg and ram percentage of 2% resulted in the lowest non-pregnant rates and highest multiple fetus rates. Non-pregnant rates and fetal number were regionally impacted, as identified by the sensitivity analysis (Table 3). Optimum reproductive potential of Maternal ewes across region and seasonal conditions was predicted to occur with a summer mating, 2% ram percentage, mating BCS 4 and mating liveweight of 60–70 kg.

The model predicted relationship between Merino ewe fetal number and each level within each node/variable is displayed in Table 6. Seasonal conditions during mating had a varied impact on non-pregnant rate as indicated by the Sensitivity analysis (Table 3). However, as conditions improved so did fetal number. A mating BCS 4, autumn mating, mating liveweight of 60–70 kg and ram percentage of 2% resulted in the lowest non-pregnant rates and highest multiple litter rates. Fetal number may be regionally impacted. Optimum reproductive potential of Merino ewes across region and seasonal conditions was predicted to occur with a summer mating, 1.5% ram percentage, mating BCS 4 and mating liveweight of 60–70 kg. 

The model predicted relationship between shedding ewe fetal number and each level within each node/variable is displayed in Table 7. Improving seasonal conditions appeared to increase fetal number, but non-pregnant rate also increased, as indicated by the sensitivity analysis (Table 3). A mating BCS 4 and autumn mating resulted in the lowest non-pregnant rates and highest multiple fetus rates. However, a mating liveweight of 90–100 kg resulted in the lowest non-pregnant rates while the greatest number of multiple fetuses was achieved at a mating liveweight 70–80 kg. Fetal number may be regionally impacted. Optimum reproductive potential of shedding ewes across region and seasonal conditions was predicted to occur with a summer mating, 2% ram percentage, mating BCS 4 and mating liveweight of 60–70 kg.

Across breeds, predicted multiple fetus rates decreased when ewe body condition at mating exceeded BCS 4 and the corresponding non-pregnant and single bearing ewe proportions increased. Similar observations were made for liveweight and ram percentage for each breed (except shedders where there was no variation in ram percentage). Taken together, the data suggests curvilinear relationships between reproduction outcomes and ewe liveweight, ewe BCS and ram percentage.

## 4. Discussion

The dataset contained little variation in pregnancy rate (90.1%) and the number of multiple fetuses (53.5%), being similar to a previous pregnancy scanning report [34]. This was reflected in the BN sensitivity analyses which indicated a low association between the variables explored, and that no standalone variable was responsible for driving pregnancy and/or number of fetuses. This highlighted the importance of considering all variables explored in this study at mating due to their interrelatedness. Although lowly associated, breed had the greatest influence on pregnancy outcome (1.35%) and fetal number (6.09%). This was expected as ovulation rates of nil, 1.62 and 2.0 in Border Leicester, between 0.2 and 1.4 in Merino and 1.0 to 1.73 in Merino cross Border Leicester ewes have previously been reported [7,8,9,35]. The influence of mating liveweight and BCS on pregnancy outcome varied by breed (Table 3). Further, mating liveweight was the most influential variable on number of fetuses in wool-based breeds and the impact of mating BCS was, again, variable across all breeds explored. The low association is likely due to the favorable seasons experienced during the survey collection period. Similarly, the influence of season on pregnancy and fetal number outcomes across the breeds varied (Table 3) and autumn was most often associated with low non-pregnant and high fetal number outcomes (Table 4, Table 5, Table 6 and Table 7). As such, the hypothesis, that the relationship between mating liveweight and BCS and pregnancy rate and fetal number vary with ewe breed and mating season, is supported.

The pregnancy rates of spring mated flocks (85%) across the survey, albeit lower than summer (90%) and autumn (93%) mated flocks, were greater than expected given the cyclic nature of the ovine reproductive cycle [1]. The length of the breeding season differs by sheep breed with cross breeds intermediate of parental potential [5,6,7,36]. This was negated in the spring mated Composite ewe flock through both a highly fecund breed and use of subcutaneous melatonin implants to advance the onset of the normal breeding cycle [23,37]. Melatonin implants, however, were only used in one other Merino flock of the remaining 11 spring mated flocks surveyed. 

Reports of ovulation and pregnancy rates outside of the breeding season are varied. The number of ovulating Merino ewes may be as low as 30% during spring in the absence of rams [8], however pregnancy rates of 80% and approximately 90% have been achieved in the presence of rams [38,39]. The ‘ram effect’, whereby reproductive hormone production, luteinizing hormone and gonadotropin releasing hormone, are stimulated by male pheromones resulting in ovulation, may be responsible for out of season mating success [40]. Twelve of the 58 ewe flocks captured (~21%) in this survey routinely practice spring mating, and must therefore, be a viable management practice regardless of the variation of reported ovulation and pregnancy rates in the literature. However, it must be noted that majority of flocks captured during spring were Merino and only two spring-mated Maternal and shedding ewe flocks were surveyed.

Further, producers ranked the season in terms of pasture availability during the mating period between ‘well below average’ to ‘well above average’, a total of five possible ranks. Over 65% of producers with spring mated flocks reported ‘above average’ to ‘well above average’ seasons. As observed previously [9,41], improved nutrition at mating may have also increased spring pregnancy rates above expected levels. 

The pregnancy rates for autumn-mated Maternal and Merino ewes in the current survey are very similar to previous reports [3,38,39,42]. Shedding breeds, such as Dorper and Damara breeds, are recognized as being non-seasonal breeders [43], which explains these results. Shedding ewes performed similarly between spring and autumn mating seasons in terms of pregnancy rate. Autumn litter size, however, was higher than spring, indicating a seasonal influence on ovulation rate in shedding breeds. Nutrition and/or genetic composition may have also influenced ovulation rate.

Pregnancy and fetal number outcomes were predicted to peak in the BN analysis at 60–69.5 kg in Composite, Maternal and Merino ewes. At liveweights lower than 60 kg and greater than 69.9 kg, pregnancy and fetal number decreased in Composite, Maternal and Merino ewes. The percentage of non-pregnant shedding ewes was least at a mating liveweight of 90–99.5 kg. Fetal number in shedding ewes, however, was greatest at a mating liveweight of 70–79.5 kg. The selection for greater fleece weight, due to the positive genetic correlation between fleece weight and bodyweight, has resulted in heavier sheep that have higher feed requirements [17,44]. However, breeding objectives for sheep producers are becoming increasingly complex [44,45] and the selection for other traits may have obscured the genetic relationship between liveweight and reproductive potential [46]. Increasing mating liveweight and BCS is known to increase conception rates and fetal number [9,10,12,47,48,49]. The Lifetime Wool guidelines recommend a mating BCS of 3, or approximately 90% of standard reference weight, at mating in Merino ewes [20,50]. These guidelines have also improved both ewe reproductive and progeny performance [51], and have been extended to Maternal ewes with a recommendation of BCS 3.8–4.2 at mating [19]. Attaining the liveweight and BCS profiles is the focus of these guidelines to optimize productivity and profitability. 

The influence of mating liveweight and BCS on conception and ovulation rate varies by breed. No improvement in conception or ovulation rate was achieved above a mating liveweight or BCS threshold in Romney, Composite and Cheviot ewes [13,14], however ovulation rate increased with mating liveweight and BCS in Beulah ewes [49]. A review of the relationship between BCS and reproductive outcomes suggested a curvilinear relationship existed between BCS and productive performance [52], as expressed in Malpura ewes, having significantly greater conception rates at mating BCS 3–3.5 compared to BCS 2 and BCS 4 [15]. A linear relationship between mating BCS and pregnancy scanned fetal number, however, was observed in both Merino and Maternal ewes in southern Australia [4]. The flocks studied were managed to have a minimum mating BCS of 2.5 and 60% of flocks were in BCS 3 or more [4]. A full range (1–5) of mating BCS was not captured and likely responsible for the linearity of the observed relationship between mating BCS and fetal number. Further, flocks of similar genetic background may respond linearly to improvements in mating BCS [53]. A curvilinear relationship was present in the current study across all breeds for pregnancy and fetal number outcomes with mating liveweight and was apparent when mating BCS was ≥4. This may be explained by the curvilinear relationship suggested between liveweight and ovulation rate [48], whereby leptin may be responsible at the follicular level for reduced ovulation rate [54]. This threshold of BCS 4 is at odds with current recommendations for Merino ewes of BCS 3 at mating [20,50], however aligns well with Maternal ewe recommendations [19]. These recommendations were based on simulated productivity and economic evaluation of liveweight profile across the production year. The greater predicted mating liveweight range in shedding breeds is likely a consequence of the low number of flocks captured. Further research into the reproductive response and management of Dorper ewes, a shedding breed, in Australia by industry has previously been recommended [55] and is still required.

The predicted impact of ram percentage on pregnancy rate and number of fetuses in the BN analysis was unexpected and was also curvilinear. Guidelines for producers recommend a ram percentage for mature ewes of 1.0–1.5% plus one additional ram [56,57]. However, the optimal ram percentage, in terms of low non-pregnant rates and high fetal number outcomes, occurred at 1.8–2.3% for Maternals, and at 1.3–1.75% in Composite and Merino breeds, which are greater than the current industry guidelines. Too few rams (less than 1%) can result in lower conception and ovulation rates, possibly due to crowding of the ram by ewes in estrus [58]. Increasing the ram to ewe ratio increased the number of times a ewe was mated, pregnancy rate and the number of twin lambs born [59,60,61]. Reasons for sub-optimal performance at high ram percentage may involve mating behaviors or nutrition. Rams may display dominance behaviors if in too close proximity, which may reduce subordinate ram performance [62]. Ewes are also reported to seek out and preference more active rams [63], which, in combination with ram dominance behaviors, may justify the lower conception and number of fetuses observed at greater ram percentages. Ram reproductive potential is also influenced by available nutrition, influencing the number of spermatozoa produced by the ram [64], however overnutrition may also reduce reproductive potential [65]. In the current study there was insufficient data collected from flocks experiencing below and well below average seasonal conditions to draw conclusions regarding ram nutrition. No further ram information was collected during the survey, however individual ram, age, libido and breed may also have had an effect [61,66].

This survey had several limitations. Capturing data across two (or multiple) years would allow the consistency of these effects to be explored as reproductive performance in the current year may be influenced by performance in the previous year [67]. Further, a relatively small number of shedding breed ewe flocks were captured. The relatively small number of flocks that received hormone treatment meant separating this data was not possible and as a result, may have unduly influenced the spring mating system, especially for the Composite breed. The use of high ram percentages may reflect low flock fertility, but greater investigation is required to fully understand the relationship. Finally, the BN analysis is specific to the survey data collected, and thus caution should be taken when extrapolating findings to a wider context.

## 5. Conclusions

The objective of this study, to examine the relationships between mating weight and BCS on reproductive success, respectively, and secondly, to explore the influence of seasonal conditions during mating and ram percentage on pregnancy and fetal number outcomes across a variety of sheep breeds and mating seasons was achieved. The BN developed is specific to the data of the sheep surveyed in this study, and, as such, extrapolating conclusions more broadly should be approached with caution. Overall, all variables explored were lowly associated with pregnancy and fetal number outcomes across this dataset and highlight the importance of understanding the interrelationships between the different mating practices and variables explored. Curvilinear relationships were observed between fetal number outcomes and mating liveweight and BCS and ram percentage. Practically, pregnancy rate and number of fetuses are predicted to vary between breeds, peaking between 60–69.5 kg in the wool-based breeds explored and between 70–79.5 and 90–99.5 kg, respectively, in shedding breeds. Further, summer-mated Composite (with no hormone manipulation) and Merino ewes with 1.3–1.75% rams and autumn mated Maternal ewes with 1.8–2.3% rams and shedding ewes (all shedding ewe data used 1.8–2.3% rams) resulted in optimum conception rate and fetal number. However, further nuances within and between breeds may be found from a larger dataset with greater flock numbers during each of the explored mating seasons and greater variation of seasonal conditions during mating. This survey study emphasizes the importance of understanding the interaction between breed, mating season, ram percentage and the influence of seasonal conditions, while ensuring an appropriate liveweight and BCS at mating.

## Figures and Tables

**Table 1:** The number of non-pregnant and pregnant ewes and fetal number (no.) by number of flocks and within each breed and mating seasons and resultant pregnancy and multiple rates of pregnant ewes. A dash (-) indicates data was absent.

Season	Breed	No. of Flocks	Pregnancy Outcome		Pregnancy Rate	No. of Fetuses		Multiple Rate
			Non-Pregnant	Pregnant		Single	Multiple	
Spring	Composite ^A^	1	17	427	0.96	36	391	0.92
	Maternal	2	134	828	0.86	301	527	0.64
	Merino ^A^	7	545	2794	0.84	1304	1490	0.53
	Shedding	2	153	845	0.85	457	388	0.46
Summer	Composite ^B^	8	298	3683	0.93	849	2229	0.72
	Maternal	8	387	3446	0.90	1215	2231	0.65
	Merino	8	462	3576	0.89	1968	1608	0.45
	Shedding	-	-	-	-	-	-	-
Autumn	Composite	7	203	4152	0.95	743	3409	0.82
	Maternal ^B^	2	213	1572	0.88	271	835	0.75
	Merino	8	188	3779	0.95	2061	1718	0.45
	Shedding	3	214	1241	0.85	477	764	0.62
Total		58	2814	26,343		9682	15,590	

^A^ One Composite and one Merino flock used Regulin^®^ ( Ceva Animal Health Pty Ltd., Glenorie, NSW, Australia), a melatonin hormone product. ^B^ Data for number of fetuses in one summer joined Composite flock and one autumn joined Maternal flock was removed from the dataset due to inaccurate scanning performance or because of a delayed date of scanning and accounts for the disparity between number of pregnant ewes and fetal number records.

**Table 2:** Bayesian network sensitivity analysis to determine how much the pregnancy outcome and fetal number (no.) nodes (variables) were influenced by each of the observed nodes. Fetal number is for positive pregnancy outcomes (‘1’ was selected in pregnancy outcome node and the fetal number (‘litter size’) sensitivity analysis performed, Figure 1). Mutual information is represented by MI and percentage by P (%). Data displayed to three significant figures (rather than decimal places).

Node	Pregnancy Outcome		Fetal No.	
	MI	P (%)	MI	P (%)
Breed	0.00616	1.35	0.0581	6.09
During	0.00225	0.493	0.0255	2.67
Mating BCS	0.00217	0.475	0.0111	1.16
Season	0.00611	1.34	0.00312	0.327
Mating weight	0.00302	0.662	0.0402	4.21
Ram	0.00425	0.931	0.00738	0.773
Region	0.00196	0.430	0.0323	3.38

**Table 3:** Bayesian network sensitivity analysis to determine how much the pregnancy outcome and fetal number (no.) nodes were influenced by each of the observed nodes within each breed, Composite, Maternal, Merino, shedding. Fetal number is for positive pregnancy outcomes (‘1’ was selected in pregnancy outcome node (variable) and the fetal number (‘litter size’) sensitivity analysis performed, Figure 1). Mutual information is represented by MI and percentage by P (%). Data displayed to three significant figures (rather than decimal places). Shaded numbers indicate the node which most influences Pregnancy outcome and Fetal number for each breed, a dash (-) indicates no data for comparison.

Node	Composite				Maternal				Merino				Shedding			
	Pregnancy Outcome		Fetal No.		Pregnancy Outcome		Fetal No.		Pregnancy Outcome		Fetal No.		Pregnancy Outcome		Fetal No.	
	MI	P (%)	MI	P (%)	MI	P (%)	MI	P (%)	MI	P (%)	MI	P (%)	MI	P (%)	MI	P (%)
During	0.00804	2.48	0.00346	0.467	0.0100	1.98	0.00962	1.06	0.0126	2.60	0.0178	1.79	0.0186	3.06	0.0531	5.36
Mating BCS	0.00107	0.331	0.00486	0.656	0.00335	0.668	0.00683	0.76	0.00184	0.381	0.0126	1.26	0.00473	0.778	0.0115	1.15
Season	0.00278	0.858	0.0109	1.47	0.00142	0.284	0.0108	1.20	0.0182	3.75	0.00403	0.404	0.0000600	0.00965	0.0174	1.75
Mating weight	0.00268	0.826	0.0230	3.10	0.00394	0.785	0.0256	2.83	0.00372	0.769	0.0398	3.98	0.00383	0.629	0.0427	4.30
Ram	0.00339	1.04	0.00919	1.24	0.0191	3.81	0.0180	1.99	0.00613	1.27	0.0106	1.06	-	-	-	-
Region	0.00765	2.36	0.00148	0.199	0.0215	4.29	0.00886	0.980	0.000690	0.143	0.0226	2.26	0.0159	2.62	0.0734	7.40

**Table 4:** The estimated probabilities for Composite fetal number (no., displayed as percentages) for each node (variable) when the different levels within each node are assumed in the Bayesian Network (BN) model, where BCS = body condition score. Level descriptions detail the data and naming conventions within each level of the model. A dash (-) indicates data were not applicable for the respective level. The BN model assessed the interrelationships between pregnancy outcome and fetal number of Composite ewes. Data displayed to three significant figures (rather than decimal places) as such the row total percentage value may be slightly different from 100% due to rounding errors. Letter prefixes are defined as: LT = less than, E = a range of values around the displayed value and GT = greater than. Region abbreviations are defined as: NSW = New South Wales, VIC = Victoria, QLD = Queensland and SA = South Australia.

Node	Level	Level Description	Fetal No.		
			Non-Pregnant	Single	Multiple
During	Well above average	Seasonal conditions described by producers during mating	10.4	23.3	66.2
	Above average		4.94	18.1	76.9
	Average		2.48	21.8	75.7
	Below average		-	-	-
	Well below average		-	-	-
Mating BCS	LT2	BCS < 2	11.2	29.7	59.1
	E2	BCS 2–2.25	7.67	25.6	66.8
	E2.5	BCS 2.5–2.75	6.36	21.3	72.4
	E3	BCS 3–3.25	5.65	21.4	73.0
	E3.5	BCS 3.5–3.75	5.22	19.3	75.5
	E4	BCS 4–4.25	5.08	16.0	78.9
	GT4	BCS ≥ 4.5	6.55	16.5	76.9
Mating season	Spring	Season of mating	3.82	8.08	88.1
	Summer		7.50	23.9	68.6
	Autumn		4.66	17.1	78.3
Mating weight	21 to 50	21 to 49.5 kg	9.05	43.2	47.8
	50 to 60	50 to 59.5 kg	6.10	25.4	68.5
	60 to 70	60 to 69.5 kg	4.53	15.8	79.7
	70 to 80	70 to 79.5 kg	5.46	15.9	78.6
	80 to 90	80 to 89.5 kg	7.02	17.9	75.1
	90 to 100	90 to 99.5 kg	7.96	17.0	75.1
	100 to 123	≥100 kg	14.1	17.2	68.7
Ram	E1	1.0 to 1.25%	4.24	13.2	82.6
	E1.5	1.3 to 1.75%	2.37	22.4	75.2
	E2	1.8 to 2.3%	5.89	18.9	75.2
	E2.5	2.5%	11.7	35.4	52.9
	GT2.5	2.8 to 4.0%	-	-	-
Region	Central NSW		7.17	19.9	72.9
	East VIC		6.90	17.9	75.1
	Northern NSW/QLD		1.73	26.0	72.3
	SA Peninsula		-	-	-
	Wimmera Mallee Murray		2.08	18.1	79.8

**Table 5:** The estimated probabilities for Maternal fetal number (no., displayed as percentages) for each node (variable) when the different levels within each node are assumed in the Bayesian Network (BN) model, where BCS = body condition score. Level descriptions detail the data and naming conventions within each level of the model. A dash (-) indicates data were not applicable for the respective level. The BN model assessed the interrelationships between pregnancy outcome and fetal number of Maternal ewes. Data displayed to three significant figures (rather than decimal places) as such the row total percentage value may be slightly different from 100% due to rounding errors. Letter prefixes are defined as: LT = less than, E = a range of values around the displayed value and GT = greater than. Region abbreviations are defined as: NSW = New South Wales, VIC = Victoria, QLD = Queensland and SA = South Australia.

Node	Level	Level Description	Fetal No.		
			Non-Pregnant	Single	Multiple
During	Well above average	Seasonal conditions described by producers during mating	7.87	25.3	66.9
	Above average		15.5	32.4	52.1
	Average		10.1	35.2	54.6
	Below average		-	-	-
	Well below average		-	-	-
Mating BCS	LT2	BCS < 2	22.7	37.5	39.8
	E2	BCS 2–2.25	16.1	34.9	49.0
	E2.5	BCS 2.5–2.75	13.1	30.7	56.3
	E3	BCS 3–3.25	10.9	30.8	58.3
	E3.5	BCS 3.5–3.75	9.51	28.5	61.9
	E4	BCS 4–4.25	9.19	24.6	66.2
	GT4	BCS ≥ 4.5	10.4	24.1	65.4
Mating season	Spring	Season of mating	13.9	31.2	54.8
	Summer		9.98	31.7	58.3
	Autumn		11.80	20.2	68.0
Mating weight	21 to 50	21 to 49.5 kg	18.5	51.8	29.7
	50 to 60	50 to 59.5 kg	13.60	36.4	50.0
	60 to 70	60 to 69.5 kg	9.79	26.5	63.7
	70 to 80	70 to 79.5 kg	9.81	25.0	65.1
	80 to 90	80 to 89.5 kg	9.93	24.4	65.7
	90 to 100	90 to 99.5 kg	9.90	21.9	68.2
	100 to 123	≥100 kg	17.7	23.1	59.2
Ram	E1	1.0 to 1.25%	30.10	25.8	44.1
	E1.5	1.3 to 1.75%	10.4	22.7	66.9
	E2	1.8 to 2.3%	8.35	24.9	66.8
	E2.5	2.5%	14.3	38.8	46.9
	GT2.5	2.8 to 4.0%	10.5	38.7	50.8
Region	Central NSW		10.1	25.9	64.1
	East VIC		4.49	38.9	56.6
	Northern NSW/QLD		12.9	36.5	50.6
	SA Peninsula		-	-	-
	Wimmera Mallee Murray		30.1	25.8	44.1

**Table 6:** The estimated probabilities for Merino fetal number (no., displayed as percentages) for each node (variable) when the different levels within each node are assumed in the Bayesian Network (BN) model, where BCS = body condition score. Level descriptions detail the data and naming conventions within each level of the model. A dash (-) indicates data were not applicable for the respective level. The BN model assessed the interrelationships between pregnancy outcome and fetal number of Merino ewes. Data displayed to three significant figures (rather than decimal places), as such the row total percentage value may be slightly different from 100% due to rounding errors. Letter prefixes are defined as: LT = less than, E = a range of values around the displayed value and GT = greater than. Region abbreviations are defined as: NSW = New South Wales, VIC = Victoria, QLD = Queensland and SA = South Australia.

Node	Level	Level Description	Fetal No.		
			Non-Pregnant	Single	Multiple
During	Well above average	Seasonal conditions described by producers during mating	13.1	35.4	51.5
	Above average		6.6	50.3	43.1
	Average		14.2	42.1	43.7
	Below average		7.24	60.9	31.9
	Well below average		20.3	62.8	16.9
Mating BCS	LT2	BCS < 2	17.7	55.8	26.5
	E2	BCS 2–2.25	12.8	54.8	32.4
	E2.5	BCS 2.5–2.75	11.3	50.2	38.5
	E3	BCS 3–3.25	9.66	50.5	39.8
	E3.5	BCS 3.5–3.75	9.05	46.2	44.8
	E4	BCS 4–4.25	9.28	39.8	50.9
	GT4	BCS ≥ 4.5	11.6	38.3	50.0
Mating season	Spring	Season of mating	16.3	38.9	44.8
	Summer		11.60	49.0	39.4
	Autumn		4.71	51.8	43.5
Mating weight	21 to 50	21 to 49.5 kg	14.2	66.0	19.8
	50 to 60	50 to 59.5 kg	9.93	55.4	34.7
	60 to 70	60 to 69.5 kg	8.33	42.3	49.3
	70 to 80	70 to 79.5 kg	9.25	39.4	51.4
	80 to 90	80 to 89.5 kg	12.10	39.1	48.8
	90 to 100	90 to 99.5 kg	15.10	34.4	50.5
	100 to 123	≥100 kg	17.9	32.1	50.0
Ram	E1	1.0 to 1.25%	14.50	62.1	23.5
	E1.5	1.3 to 1.75%	11.4	42.7	46.0
	E2	1.8 to 2.3%	8.60	46.5	44.9
	E2.5	2.5%	18.9	50.8	30.2
	GT2.5	2.8 to 4.0%	16.3	51.3	32.4
Region	Central NSW		9.82	42.4	47.8
	East VIC		-	-	-
	Northern NSW/QLD		10.4	60.6	29.0
	SA Peninsula		12.5	50.5	37.0
	Wimmera Mallee Murray		11.8	39.3	48.8

**Table 7:** The estimated probabilities for shedding fetal number (no., displayed as percentages) for each node (variable) when the different levels within each node are assumed in the Bayesian Network (BN) model, where BCS = body condition score. Level descriptions detail the data and naming conventions within each level of the model. A dash (-) indicates data were not applicable for the respective level. The BN model assessed the interrelationships between pregnancy outcome and fetal number of shedding ewes. Data displayed to three significant figures (rather than decimal places), as such the row total percentage value may be slightly different from 100% due to rounding errors. Letter prefixes are defined as: LT = less than, E = a range of values around the displayed value and GT = greater than. Region abbreviations are defined as: NSW = New South Wales, VIC = Victoria, QLD = Queensland and SA = South Australia.

Node	Level	Level Description	Fetal No.		
			Non-Pregnant	Single	Multiple
During	Well above average	Seasonal conditions described by producers during mating	-	-	-
	Above average		18.0	28.6	53.5
	Average		4.80	50.8	44.4
	Below average		-	-	-
	Well below average		15.8	59.0	25.1
Mating BCS	LT2	BCS < 2	26.9	44.4	28.6
	E2	BCS 2–2.25	19.9	43.8	36.3
	E2.5	BCS 2.5–2.75	17.2	39.8	42.9
	E3	BCS 3–3.25	14.6	40.7	44.7
	E3.5	BCS 3.5–3.75	13.0	37.5	49.5
	E4	BCS 4–4.25	12.1	31.7	56.2
	GT4	BCS ≥ 4.5	12.4	31.8	55.8
Mating season	Spring	Season of mating	15.3	45.8	38.9
	Summer		-	-	-
	Autumn		14.70	32.7	52.6
Mating weight	21 to 50	21 to 49.5 kg	21.3	57.5	21.1
	50 to 60	50 to 59.5 kg	16.4	45.3	38.3
	60 to 70	60 to 69.5 kg	13.4	33.1	53.5
	70 to 80	70 to 79.5 kg	14.3	28.5	57.2
	80 to 90	80 to 89.5 kg	12.2	34.0	53.8
	90 to 100	90 to 99.5 kg	9.80	35.0	55.2
	100 to 123	≥100 kg	10.4	30.5	59.1
Ram	E1	1.0 to 1.25%	-	-	-
	E1.5	1.3 to 1.75%	-	-	-
	E2	1.8 to 2.3%	14.90	38.0	47.0
	E2.5	2.5%	-	-	-
	GT2.5	2.8 to 4.0%	-	-	-
Region	Central NSW		14.4	37.0	48.5
	East VIC		-	-	-
	Northern NSW/QLD		9.69	54.5	35.9
	SA Peninsula		-	-	-
	Wimmera Mallee Murray		24.0	14.1	61.9

## Data Availability

The data that support this study cannot be publicly shared due to ethical or privacy reasons and may be shared upon reasonable request to the corresponding author if appropriate.
